# Supplementary material for: Linear Mixed-Effects Model to Quantify the Association between Somatic Cell Count and Milk Production in Italian Dairy Herds
Source: Animals (Basel). 2022 Dec 25;13(1):80. doi: 10.3390/ani13010080 (PMC9817942; doi:10.3390/ani13010080)
Supplement: Supplementary file 1 [file animals-13-00080-s001.zip › animals-2093994-supplementary.pdf]

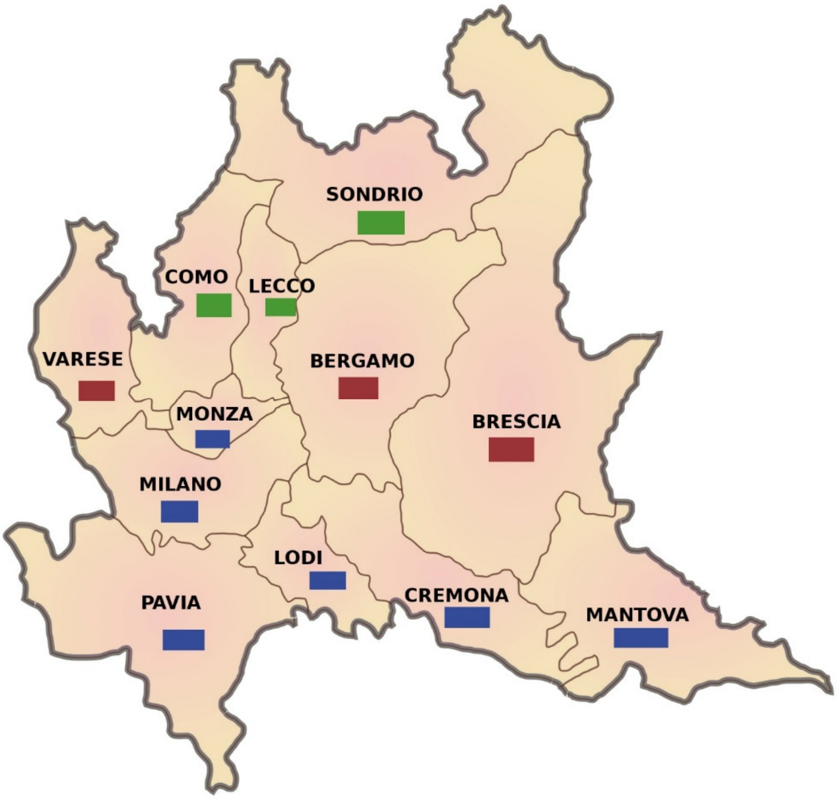

Figure S1. The 12 provinces of Lombardy as classified in the paper: the blue color represents the provinces classified in the Po Valley group; the red color represents the provinces classified in the Semi-alpine group and the green color represents the provinces classified in the Alpine group.

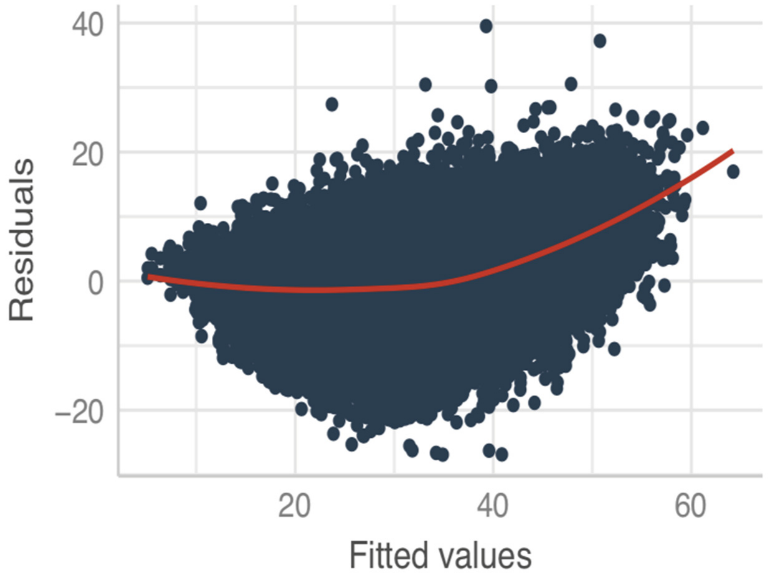

Figure S2. Standard residuals vs fitted values from the linear mixed model to estimate the association between SCC and milk production (10,445,464 test-day records).

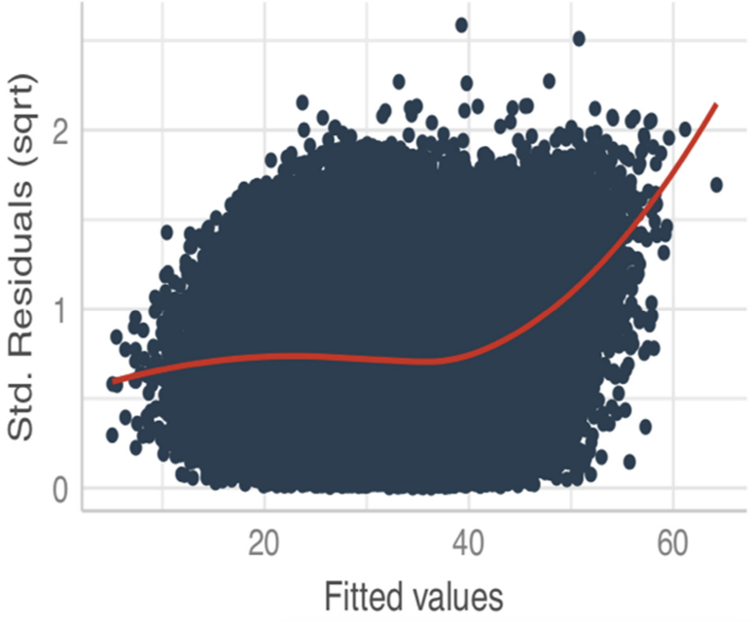

Figure S3. Scale-Location plot from the linear mixed model to estimate the association between SCC and milk production (10,445,464 test-day records).
